# Image-Based Monitoring of Thermal Ablation

**DOI:** 10.3390/bioengineering12010078

**Published:** 2025-01-15

**Authors:** Xinyi Wang, Shiqing Zhao, Aili Zhang

**Affiliations:** School of Biomedical Engineering, 400 Med-X Research Institute, Shanghai Jiao Tong University, 1954 Huashan Road, Shanghai 200030, China; wangxinyi_anthea@sjtu.edu.cn (X.W.); shiqingzhao@sjtu.edu.cn (S.Z.)

**Keywords:** thermal therapy, imaging technology, temperature monitoring, physical property changes

## Abstract

Thermal therapy is a commonly used local treatment technique in clinical practice. Monitoring the treatment process is essential for ensuring its success. In this review, we analyze recent image-based methods for thermal therapy monitoring, focusing particularly on their feasibility for synchronous or immediate postoperative monitoring. This includes thermography and other techniques that track the physical changes in tissue during thermal ablation. Potential directions and challenges for further clinical applications are also summarized.

## 1. Introduction

Thermal ablation is a localized therapeutic technique widely used in clinical practice. It applies thermal energy to targeted areas within the body to induce cell necrosis or apoptosis, aiming to treat diseases such as malignant tumors or to alleviate symptoms associated with conditions like neuropathic pain and atrial fibrillation [1]. Since the first application of percutaneous radiofrequency ablation in clinics in 1989 [2], it has become a standard treatment for solid tumors in various sites, including the liver, lungs, kidneys, and breast [3]. With advances in tomographic imaging techniques like CT, MRI, and ultrasound, image-guided percutaneous thermal ablation is now considered a first-line therapy for the radical treatment of small tumors and the conservative care of inoperable tumors [3]. In addition to utilizing thermal energy alone for treatment, thermal therapy is often recommended to be combined with other modalities, such as radiotherapy, chemotherapy, or gene therapy [4]. Thermal energy not only induces cytotoxic effects but also triggers antitumor immune responses, disrupts the tumor vasculature and thus blood supply, changes cell membrane integrity, deactivates DNA and proteins, and alters metabolic processes, et al. [5]. Thermal ablation has evolved beyond merely managing energy focus and ensuring tumor coverage [6] to actively modulating biological responses, enhancing both localized and systemic antitumor effects [7].

The therapeutic effects induced by heat are not only determined by the treatment temperature but also by the biological system itself [5]. Effective therapy of thermal treatments comes from accurate energy delivery, but also precise monitoring of the biological response, which is closely related to the monitoring ability throughout the process.

**Figure 1:**
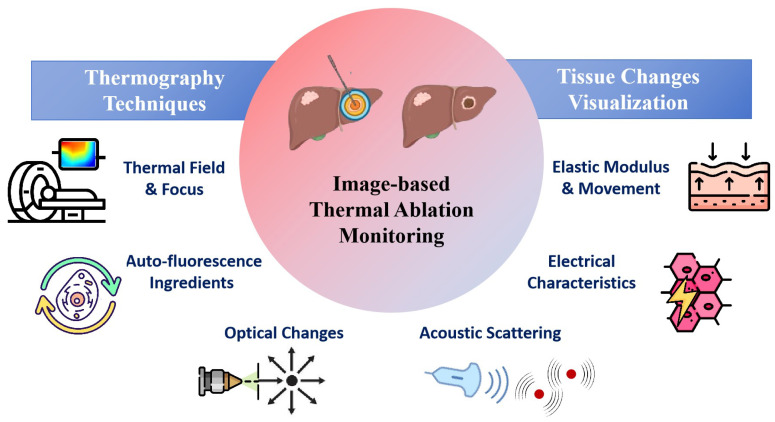
Image-based Thermal Ablation Monitoring Techniques.

In this review, we have reviewed recent studies on image-based monitoring techniques for thermal therapy, with a focus on real-time intraoperative or immediate postoperative monitoring method, as shown in Figure 1. The monitoring techniques are categorized into temperature imaging and thermal damage imaging, with an emphasis on thermal damage related changes in physical properties of tissue. The potential directions and challenges for clinical applications were also addressed.

## 2. Thermography Monitoring

**Figure 2:**
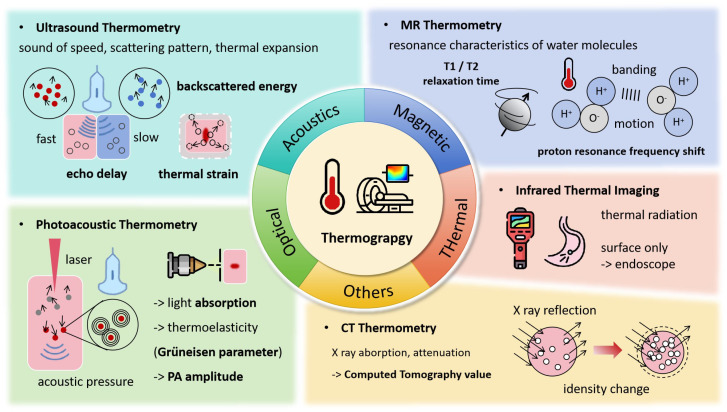
Thermograpy Monitoring Techniques and Associated Signal Changes.

Temperature serves as a key indicator of heat application, as shown in Figure 2, with various forms of energy—such as radiofrequency, microwaves, HIFU, and lasers—being absorbed by tissues and converted into heat, leading to a series of irreversible injuries. Accurately obtaining temperature fields and thermal history is essential for adjusting the energy level for effective treatment. The direct use of temperature field isotherms enables rapid estimation of the extent of thermal damage to tumors. Continuous monitoring of thermal processes is just as crucial as understanding thermal distribution. Ideal thermometry technology should meet several criteria [8]: temperature accuracy within 1–2 °C across the relevant range, 3D spatial resolution of less than 1–2 mm, acquisition times under 10–30 s, and real-time display capabilities. Currently, several thermography techniques are used to monitor the thermal ablation process, including MRI, CT, ultrasound, and others.

### 2.1. MR Thermometry

Measurable MR parameters, including diffusion coefficients, T1 and T2 relaxation times, magnetization transfer, proton density, and proton resonance frequency shift (PRFS) [9,10,11], are all temperature-dependent and have been used for temperature imaging, with PRFS being the most widely utilized. MRI PRFS-based temperature measurement does not require pre-calibration, as the PRFS signal remains consistent across different tissue types and thermal conditions [12]. This technique exhibits a strong linear correlation with temperature changes within the range of −15 °C to 100 °C.

In vivo studies on laser thermal treatment of porcine limb muscle have reported the largest temperature measurement error of 1.4 °C, a temporal resolution of 8 Hz, and an in-plane resolution of 1.4 × 1.4 mm with a 3 mm slice thickness using 2D fast gradient echo of MRI PRFS [7]. When using 3D Fast Gradient Echo sequence offline, an accuracy of 0.20 °C in phantom and 0.75 °C in brain tissue during microwave mild heating was achieved with a sacrifice of both scanning rate and spatial resolution [13]. To improve MRI thermometry accuracy in the abdomen and chest, where motion artifacts are common [14], Valéry Ozenne et al. combined multi-slice Echo Planar Imaging sequences with 3D motion correction, obtaining volume temperature imaging with errors below 2 °C during RFA treatment of the liver under respiratory motion [15]. A Double Echo Planar Imaging pulse sequence was also used to minimize the impact of uneven magnetic fields, achieving an accuracy of 0.75 °C for RFA treatment of brain tissue [16]. To address the challenge of low frame rates when capturing rapid temperature changes, a block-based image compression method was proposed [17]. This method, with a 7:1 compression ratio, keeps a temperature reconstruction error of under 2.5 °C.

Due to its absolute temperature measurement ability, MR thermometry has been integrated with thermal treatment systems such as HIFU to achieve automatic ablation process control with feedback from MR measurements [18]. The integrated MR thermometry technique also helped locate the HIFU focus in vivo [18]. An algorithm to correct the perturbation on the MRI signal from the microwave radiation was found necessary when MRI thermometry is used during microwave ablation [19].

### 2.2. Ultrasound Temperature Measurement

As temperature increases, the speed of sound also rises. Additionally, as tissues undergo thermal expansion during heating, their acoustic properties (such as acoustic impedance) change, which affects the pattern of sound scattering. The parameters currently used for ultrasound thermometry include frequency-domain attenuation, change of backscattered energy (CBE), and echo time-shift [9,10], among others.

The backscattered power [20,21] and harmonic amplitude [22] of ultrasound have been shown to be temperature-dependent. As the temperature increased from 26 °C to 46 °C, significant signal enhancement was observed in the amplitudes of the fundamental frequency, second harmonic, and third harmonic, as well as in their ratios, in ex vivo bovine muscle tissue [23]. A second harmonic CBE-based method has been used to control localized heating in low-intensity focused ultrasound (LIFU) [22,24]. Within the 37–47 °C range, the focal temperature was maintained for 6 min with an error of less than 0.6 °C [24]. In a water bath heating experiment from 36 °C to 45 °C, the temperature estimation accuracy was within 0.54 °C, meeting the requirements for HIFU therapy [25].

For temperatures above 50 °C, when thermal expansion starts to impact the acoustic linear dependence on temperature, the adaptively adjusted temperature measuring error by the echo time shift method was reduced to 6% from 50% [26]. Unlike directly linking temperature with sound parameters, the ultrasound thermal strain imaging (TSI) method calculates the thermal strain of tissues by analyzing the time and phase shifts of ultrasound echo signals, thereby indirectly deriving the temperature change of the tissue [27,28]. In a recent study, for a temperature rise of 45 °C in heart tissue ablation (from 25 °C to 70 °C) within 30 s, the TSI method estimated a temperature increase of 46 °C [29].

Deep-learning-based approaches have been proposed to enhance ultrasound (US) temperature measurement accuracy in thermal therapy. By integrating both the US backscattering data with heat transfer physics into the machine learning algorithm, temperature measurement within the HIFU focus achieved an accuracy of 0.5 °C [30]. Additionally, by using the temporal echo shift US data and temperature history as inputs for a neural network model, a maximum absolute error of 0.5 °C was achieved in HIFU treatment of phantoms. With fewer neural units, the model also demonstrates its potential for real-time monitoring [31].

### 2.3. Photoacoustic Temperature Measurement

Compared to ultrasound, photoacoustic imaging offers a significant improvement in spatial resolution, reaching scales of hundreds of micrometers [10]. In photoacoustic temperature measurement, a laser beam is absorbed by the tissue, inducing thermal expansion and generating acoustic signals. These acoustic waves travel through or along the surface of the tissue and are detected by ultrasound sensors. The temperature distribution within the tissue is then inferred from these acoustic signals, as they are temperature-dependent.

Schüle et al. demonstrated a linear correlation between the amplitude of the photoacoustic (PA) signal (the generated acoustic wave) and temperature for temperature changes ranging from 30 °C to 80 °C [32]. In the laser treatment of retinal, for a temperature rise of 7 °C in the laser focus, the PA estimation error is 1 °C, while for a temperature rise of 60 °C, the error reaches 11 °C. With the assistance of a thermocouple for point temperature measurement, PA imaging successfully generated the absolute temperature distribution in deep tissues with an accuracy of 0.8 °C, achieved within a scanning time of 10 min [33]. Using two laser beams of different wavelengths and nanomaterials to enhance the signal, PA temperature measurements in phantoms reported an accuracy of 0.3 °C at 26 °C [34]. Furthermore, with a new light source, Paul et al. introduced a PA temperature controlling system with a time resolution of 1 ms and a sensitivity of 0.31 °C [35].

The Grüneisen parameter, which is critical in photoacoustic imaging as it links the thermal expansion induced by light absorption to the resulting acoustic pressure, was treated as a constant across the entire temperature range [36]. However, this parameter is tissue- and temperature-dependent [34]. To address this issue, Lee et al. utilized gold nanoparticles, enabling successful temperature measurements in different tissue backgrounds [37]. Additionally, a novel approach known as thermal energy memory-based photoacoustic temperature (TEMPT) was introduced to tackle this limitation [38]. TEMPT enabled successful temperature mapping during HIFU therapy in a mouse model at a depth of 2 mm, achieving an accuracy of 0.9 °C within the temperature range of 20 °C to 55 °C [39].

Photoacoustic temperature measurement has been successfully integrated into the optical path of laser ablation and photo-thermal therapy systems, enabling real-time temperature recording at the laser focus during treatment. The measurement accuracies of these systems range from 0.41 °C to 0.84 °C [40,41]. Furthermore, a laser ablation system that incorporates a Proportional-Integral-Derivative (PID) algorithm, along with photoacoustic signal-based temperature feedback, has been developed to efficiently control the extent of photo-thermal damage, enhancing the precision and safety of the therapy [42].

### 2.4. CT Thermometry

As different tissue ingredients exhibit unique X-ray absorption characteristics, changes in CT values during thermal therapy can serve as reliable indicators of temperature variations. This technique offers a spatial resolution of 1.2 mm, a temporal resolution of 2 Hz, and a temperature measurement error between 3 and 5 °C in ex vivo tissue, across a wide range of 20 °C to 100 °C [43]. However, for in vivo clinical applications, the temperature measurement error of CT imaging has been reported to range from 2.7 °C to 16.2 °C during RFA treatment [44].

Concerns about ionizing radiation exposure have significantly limited the application of CT-based thermal tomography. To address this, a low-dose CT reconstruction algorithm utilizing deep learning has been proposed [45]. The integration of AI has further improved the accuracy of temperature measurement. Wang et al. utilized photon-counting CT’s ability to differentiate between particles of varying energy levels [46]. With a neural network model, temperature measurement precisions of 3.97 °C in ${\mathrm{CaCl}}_{2}$ solution and 1.8 °C were achieved, making it a promising tool for more versatile and safer thermal field monitoring in clinical settings.

In addition to MRI, CT, and US thermometry techniques, there have also been studies on OCT temperature measurement under development [47,48]. These newly developed techniques offer additional options for temperature monitoring during thermal treatment.

## 3. Tissue Property Changes Monitoring

**Figure 3:**
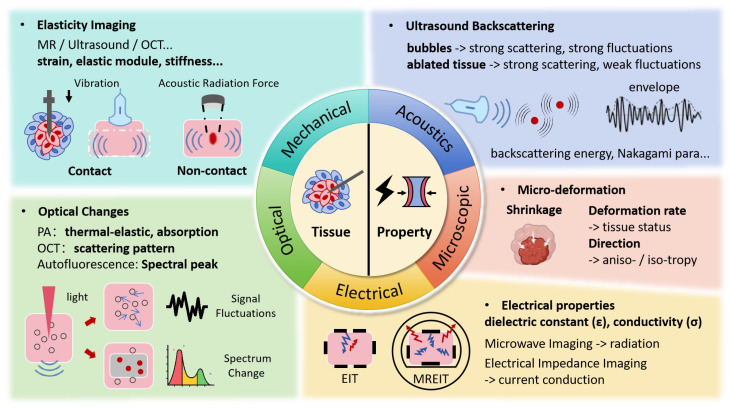
Ablated Zone Tomography Monitoring Techniques and Associated Signal Changes.

While temperature serves as an important indicator of thermal injury, it does not directly reflect the inherent tissue changes. When exposed to lethal thermal energy, biological tissues undergo a range of transformations, including protein denaturation, cell death, tissue coagulation, and vasculature rupture, among others [5]. However, due to the delayed effects of heat, the induced structural changes are typically observed as stable imaging results at least 10 min to 1 h after the procedure [2,49], making it unsuitable for intraoperative tracking. As shown in Figure 3, a more promising approach is to monitor these effects through changes in mechanical, optical, and electrical properties, and other related factors, which can be captured during therapy.

### 3.1. Mechanical Property Changes

The changes in the mechanical properties of cancerous tissue after thermal ablation can serve as valuable indicators of thermal damage [50]. Protein denaturation is the primary mechanism driving cell death in ablative therapies [51,52]. Structural proteins like collagen and elastin, which are key components of the extracellular matrix in muscles, connective tissues, and cancers are affected. When collagen denatures, its helical structure unravels, changing its mechanical properties [53]. Studies show that protein denaturation and tissue dehydration from heating significantly affect tissue elasticity [54]. During and after radiofrequency ablation, the stiffness of myocardial tissue significantly increases [52].

Ultrasound imaging can be used for elastography, with studies demonstrating strong correlations between elasticity imaging and pathological analysis of lesions [55,56]. In animal studies and clinical trials, thermal lesion boundaries were clearer in ultrasound elastography imaging compared to standard B-mode imaging [57,58]. A clinical trial involving 25 liver RFA patients showed that elastography was highly effective, with significant correlations between lesion dimensions (long axis, short axis, and area) and elastographic imaging. The correlation coefficient (R) between the dissected tissue’s color change in vitro and the region showing noticeable elasticity changes in elastography images reached 0.93–0.97 [55,56]. However, factors such as tissue motion and uncertainty in elasticity thresholds due to tissue coagulation can lead to lesion size underestimations compared to in vitro studies [59], with the correlation coefficient dropping to 0.8–0.81 [57]. Katsutoshi Sugimoto et al. introduced a 3D Shear Wave Elastography method with 80% accuracy in real-time liver ablation volume assessment during RFA [60]. Recent approaches integrating tissue biomechanical models, tissue deformation estimation, and optimization methods have further improved lesion prediction accuracy to over 90% [61]. Additionally, the time-reversible thermoacoustic elastography developed by N. Benech has been reported to work better for non-homogeneous tissues [62].

Magnetic resonance elastography (MRE), using either contact-based or non-contact vibration excitation, can assess elasticity changes during the ablation process without waiting for delayed biological responses [2]. By using external actuators or ablation probes to induce mechanical perturbations, enhanced elasticity imaging contrast was observed in the coagulation zones of porcine and bovine tissues post-ablation [63]. During liver laser treatment, MRE imaging obtained with an inertial driver integrated with the laser catheter detected significant increases in tissue stiffness, ranging from 22.5% to 64.4% [64]. Choquet et al. used offline calculations to assess anisotropic mechanical property changes in muscle tissue during HIFU treatment [65]. Corbin et al. introduced an interventional MRE system that updates 2D elasticity maps every 2.56 s, effectively monitoring stiffness changes during in vivo porcine liver ablation [66]. In addition to external actuators and ablation probes, acoustic waves are used as non-contact elastic excitation in MR acoustic radiation force imaging (MR-ARFI), providing precise control in elasticity measurements [67,68]. During ex vivo porcine liver HIFU ablation, the stiffness contour of the ablation zone aligned with CEM43 isodose lines, indicating a relationship between mechanical changes and thermal injury [69]. Deep learning techniques have accelerated the MRE acquisition rate, further improving real-time monitoring capabilities during thermal ablation [70].

Other than ultrasound and MRI elastography, other stereography techniques have been developed, such as PA elastography [71,72]. OCT elastography is also applicable for monitoring mechanical property changes during thermal ablation. However, while the post-heating analysis of the strain and stiffness dynamic changes of collagen tissue in laser ablation [73] and elastic changes in liver and cancer thermal ablation demonstrate the feasibility of OCT in detecting the thermal ablation zone with a higher resolution [74], real-time imaging for monitoring the process is yet to be developed.

Specific thresholds of the physical parameters’ change help quantify the spatial distribution of pathological damage, though they vary by tissue and patient [2]. After radiofrequency ablation, the average elasticity of the porcine liver increased from 6.4 ± 0.3 kPa to 38.1 ± 2.5 kPa, and if using a threshold of 20 kPa, 83% of the necrotic areas can be correctly identified [60]. An elasticity threshold of 48–50 kPa was used to delineate the ablated zones of the rabbit liver, with an accuracy of 88% [75]. While for the mathematical models directly defining property changes with the thermal damage, there are very limited studies. In an ex vivo heating experiment of bovine liver and muscle tissues, the shear modulus measured by ultrasound demonstrated significant temperature-dependent variation patterns [76], which can be divided into four phases: the shear modulus linearly decreased with temperature up to 43 °C (phase 1), with a change in slope at 37 °C (phase 2), then exponentially decreased up to 57 °C (phase 3), and finally exponentially increased until the end of the heating process (phase 4). The time–temperature slope measured through tissue stiffness was found to be identical to the cell death measurements reported in the original thermal dose study [77]. Through ultrasound measurement of the alpha parameter in a tissue viscoelastic model, polynomial fitted equations describing the change of the elastic modulus and relaxation time constant in relation to the thermal dose were provided [78].

### 3.2. Ultrasonic Backscattering Changes

The variation in acoustic properties caused by heating-induced changes in tissue composition and structure was revealed through the backscattered ultrasound signals [79]. As temperature rises beyond a certain threshold [20,21], backscatter power increases in an almost logarithmic manner. With the frequency-domain backscattered energy change ultrasound imaging, a strong spatial correlation (r = 0.97) and a 2.5-fold increase in image contrast (4 dB) were reached when tracking the high-intensity focused ultrasound (HIFU) focal zone [80].

The dynamic change of the Nakagami parameter derived from the envelope of the backscattered ultrasound signal, which characterizes the statistical properties of the scattering, was found to effectively reflect the thermal damage induced by HIFU and microwave ablation [81,82,83]. After HIFU and microwave ablation, the Nakagami parameters for bovine liver increased by 1.4 times (from 0.72 to 1.01) in vitro and 1.33 times (from 0.54 to 0.72) in vivo [81]. Zhang et al. found that the Nakagami parameter increased with bubble formation, then decreased after heating stopped, and stabilized at a higher value due to irreversible tissue changes [83]. The correlation coefficient R of the ultrasound Nakagami image high signal region to the histologically identified lesion is higher than 85% in vivo [84]. Additionally, real-time monitoring based on Nakagami imaging could be more effective when elastography is unavailable or when substantial bubbles are present in the ablation zone [82,85].

### 3.3. Optical Changes

Based on thermally induced optical adsorption change, PA imaging differentiates three different regions: highly coagulated, mildly coagulated, and healthy tissues [86]. Using multi-wavelength photoacoustic (MWPA) imaging, it was observed that the absorption of a 760 nm laser in tissue disappeared after ablation [87], while a lesion prediction accuracy of 70% was achieved in HIFU treatment of myocardial tissue [36]. The MWPA signal was also found to correlate with changes in hemoglobin, myoglobin, and protein denaturation levels [88]. Rebling et al. developed an integrated catheter system for RF ablation with real-time 3D PA imaging at 10 Hz, which revealed a uniform coagulation zone with irregular boundaries [89]. By minimizing the number of wavelengths required in MWPA imaging, AI algorithms enable real-time monitoring of tissue optical absorption changes during thermal treatments [90].

Optical coherence tomography (OCT), with its micrometer-level resolution, is also employed to monitor changes in optical properties during thermal therapy. OCT signals, including both the amplitude [47,48,91,92,93] and the phase [94,95,96,97] of backscattered light, correspond to tissue changes induced by heating. The fluctuation of the OCT speckle pattern is related to heating temperature and tissue state [47]. During the process of egg white heating with a water bath, an increase in speckle variance was observed, corresponding to three stages: liquid, solid-liquid mixture, and fully solid [48]. Lo et al. monitored laser ablation based on the complex differential variance (CDV) of OCT [95]. During 10 s of esophageal endoscopic radiofrequency ablation, CDV achieved 95% pathological consistency within a 2 mm imaging depth [94]. With Doppler OCT, Müller et al. distinguished the reversible thermal expansion phase from the irreversible tissue changes during retinal laser ablation [97]. The polarization of OCT is sensitive to the birefringence of muscle fibers, and real-time monitoring and feedback systems for myocardial RFA have been developed [98].

Spontaneous fluorescence hyperspectral imaging (HSI) has been used to visualize RFA lesions in heart tissue based on changes in the endogenous fluorescence spectra [99,100,101] related to the levels of NADH (indicating redox activity), hemoglobin (blood flow related), and fiber protein, with lesion size measurement error within 0.5 mm compared with histological findings [102].

### 3.4. Electrical Property Changes

Using pairs of electrodes that deliver high-frequency electrical currents and detect potential changes, impedance imaging can capture electrical property variations or impedance shifts, which correlate with tissue temperature and lesion formation [103,104,105,106,107]. The electrical impedance tomography (EIT) method estimates the damage area, with an 11.5% error compared to the real coagulative necrosis zone in bovine liver following radiofrequency ablation (RFA) [108]. Besides, integrating the impedance values obtained from EIT enables real-time control of ablation depth [109] and adjustment of the thermal field [110].

Recent advances in electrical impedance imaging have moved beyond traditional electrode-based methods. Emerging techniques such as magnetic resonance electrical impedance tomography (MR-EIT), acousto-electric imaging (AEI), Lorentz force electrical impedance tomography (LF-EIT), and magneto-acoustic tomography (MAT) combine ultrasound, magnetic fields, and electrical signals, offering enhanced resolution [111], comparable to MRI and ultrasound, without solely relying on increasing the number of electrodes.

## 4. Discussion

Compared to hyperthermia (40–45 °C for hours), ablation involves higher temperatures (>60 °C) and shorter durations (seconds to minutes) [112]. Ablation procedures are typically localized treatments that directly target lesions with concentrated energy, rather than whole-body or broad-region thermal exposure in hyperthermia [112]. Tissue changes during ablation occur rapidly and irreversibly, requiring precise control for treatment efficacy and safety. Thus, intraoperative real-time monitoring and immediate post-treatment confirmation are particularly important for thermal ablation therapy.

Sensors like thermocouples, Bragg grating optical fibers, and other emerging technologies have also made great progress in measurement accuracy, miniaturization, and electromagnetic compatibility [113]. These temperature sensors and impedance measurements are being used in clinical applications [114,115], providing discrete point measurements [116]. As the in vivo treatment range is three-dimensional and non-uniform, spatial temperature mapping provides additional information, particularly for treatments near sensitive structures [117]. The ability to non-invasively map temperature distributions across the entire treatment volume enables more precise control of thermal damage boundaries [118,119], which is important for protection of critical tissues while ensuring complete treatment coverage.

With the thermography techniques based on MRI, ultrasound, or CT modality, both the distribution and the dynamics of temperature in the targeted tissue can be obtained. They have significantly improved the ability to monitor thermal therapies. For example, MR thermometry-guided ablation has demonstrated unique advantages in neurosurgical applications [120] and hepatic tumor treatments [121] by enabling precise control of thermal damage boundaries. However, these techniques still face limitations, particularly in terms of temperature range and accuracy. The complex biological changes that occur at elevated temperatures—such as cell death, water evaporation, and protein denaturation, et al. interfere with the precision of temperature measurements [26,47,122]. While current methods offer valuable insights into the temperature-changing dynamics, they are often limited by the challenges of detecting temperature variation across a broad spectrum of tissue changes, limiting their overall effectiveness in certain clinical scenarios [9]. Besides, although image acquisition [95,108] and post-processing [90] are rapid and show potential for real-time applications, most findings are from offline intra-operative data processing.

The heating method’s interference on temperature imaging is an important issue. It depends on both the thermography technique and heating methods. MR thermometry, the clinically approved method in HIFU and LITT treatments [11], allows temperature to be continuously monitored during heating with minimal influence. However, it cannot be used with non-compatible probes of RFA and MWA [123,124]. During HIFU procedures, ultrasound temperature measurement often encounters interference since the monitoring and treatment energy operate within the same physical field [10,125]. The electromagnetic interference [123], heating-induced optical and acoustic fluctuations [92,126], and breathing [127] further affect the accuracy of different temperature imaging techniques. Correction algorithms have been developed to decrease the possible inferences [38,128,129].

The direct application of temperature field isotherms allows for a rapid and accurate estimation of the thermal damage region. When combined with thermal damage models—such as temperature thresholds, CEM43 thermal dose, and Arrhenius thermal damage [130,131,132]—these temperature measurements can precisely predict the extent of localized damage [133,134], thereby offering enhanced control over treatment outcomes. For example, integrating MRI thermography into automated ablation systems has greatly improved the treatment precision of HIFU [18], laser [133], and magnetic hyperthermia [135]. The thermal focus can be controlled via PID feedback [42], achieving 0.72 °C accuracy in photothermal therapy using a photoacoustic-ultrasound dual-mode system [41]. Though an error of 1–2 °C may lead to a 3–5 fold treatment time difference for hyperthermia [136], for thermal ablation with short duration and high temperature, the MR thermometry error of around 1 °C is acceptable [132]. Studies have shown that with cell death prediction models based on temperature–time history for thermal ablation, a 1–2 °C difference means several hundreds of micrometer change in treatment range [137], which is negligible comparing to the size of the tumors (usually centimeters) [132].

**Figure 4:**
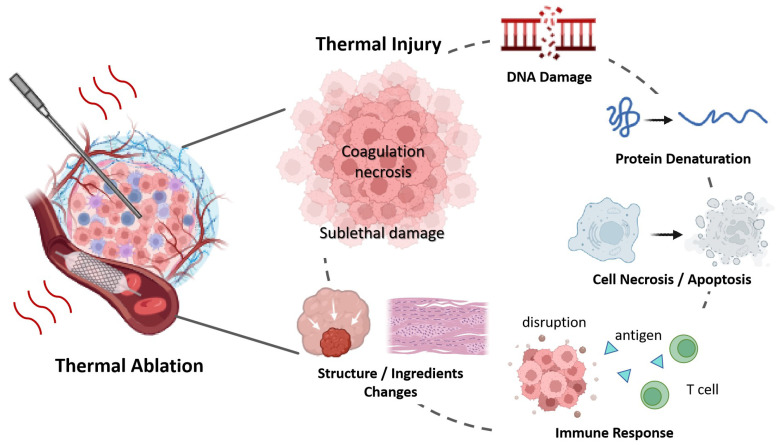
Tissue Changes During Thermal Ablation Therapy.

While temperature metrics are essentially indirect measurements of thermal damage [138], they do not provide direct information about tissue inherent changes [50]. As shown in Figure 4, thermally induced injury includes the denaturation of protein, DNA, collagen, cell death, blood flow changes, and alterations in tissue structure, et al. The above review confirmed that the physical changes—across mechanical, electrical, and optical domains—can serve as indicators of thermal injury, helping to assess the effectiveness of treatments and ensuring precision in ablative therapies.

However, thermal injury to biological systems is a dynamic process. As shown in motion tracking [139], elastography [52,140], and electrical impedance [108] monitoring, tissue continues to change after ablation. Damage to the tissue may continue due to the blood flow shutdown, progression of delayed cell death (apoptosis), or even immune responses, et al. [5]. Depending on the dosage or individual response, the cells or tissue exposed to the energy may recover from the trauma when the repairing mechanism is triggered [100]. Therefore, intraoperative damage visualization may underestimate or overestimate the treatment boundaries [94]. Further studies of the above-developed imaging techniques, together with further biological analysis, may help establish methods for actually monitoring the final therapeutic outcome other than acute tissue injury.

Other than the reviewed techniques in monitoring the thermal ablation process, there are a couple of new techniques in development. Given the observed phenomena during thermal treatment by CT, including the tissue contraction [141] of up to 45% in MWA, mild tissue-specific expansion before contraction [139], anisotropic contraction in the carbonization zone [142], and the multivariate relationship between tissue contraction and heating temperature [143], the movement of tissue during thermal ablation is also promising for developing new detection methods to monitor lesion formation in real time. Microbubble formation dynamics is another indicator, though it only appears when water evaporation occurs [144]. The microwave propagation in tissues, which is mainly influenced by water content, has been traced through microwave radiation imaging with multiple specially designed antennas positioned near the heating source in deep tissue [145,146,147].

The fast development of artificial intelligence (AI) has significantly enhanced the monitoring and control of treatments. Neural networks have identified complex temperature rise patterns from raw ultrasound signals [30], with measurement errors below 0.5 °C. The tissue-dependence limitations can also be reduced [46]. As handling motion interference and scattering noise better [148,149], these methods produced smoother, higher-contrast images and enabled rapid estimation of damage area with 96.26% accuracy [90]. AI helps recover high-quality images from sparse data, such as downsampled MRI [70] and low-dose CT [45], advancing real-time monitoring. Combined with techniques like photoacoustic (PA) imaging, the deep learning framework helps reveal biological correlations in thermally induced damage [150].

Multi-modal approaches offer further advancements. By integrating these diverse changes—mechanical, electrical, and optical—into a unified damage indicator, clinicians can achieve a more comprehensive and accurate assessment of thermal damage, potentially aligning better with histological analysis and improving the control and outcomes of thermal therapies. Structural and functional imaging have been combined, such as ultrasound B-scan with photoacoustic thermometry or damage estimation [151,152], and MRI with its elastography based on acoustic radiation force [67]. Some studies also explore the integration of multiple functional imaging channels, like IR thermometry with microwave imaging [146], and photoacoustic thermometry with OCT for ablation zone estimation [153]. When the detection and treatment energy sources overlap, integrated diagnosis and therapy are possible, as seen with HIFU therapy and ultrasound thermometry [154], and laser ablation with OCT elastography [73]. These advancements provide a more comprehensive visualization of tissue changes during treatment. With the help of the AI algorithm, which is inherently well-suited for rapidly processing high-throughput information across multiple channels, and combined with real-time closed-loop spatial and temporal control, the treatment outcome can be optimized with conformal treatment come into true [155].

## 5. Conclusions

Recent advances in thermal therapy monitoring have markedly enhanced the accuracy of treatment. MRI, ultrasound, and photoacoustic imaging-based thermography provide essential information on temperature distribution during ablation procedures. Beyond temperature measurement, modern techniques capture diverse changes in physical properties—mechanical, electrical, and optical—that reflect complex tissue responses to therapy. Technologies such as electrical impedance tomography, optical coherence tomography, and ultrasound elastography enable detailed assessments of variations in density, elasticity, and structural integrity, offering a comprehensive, multidimensional perspective on treatment effects. The integration of artificial intelligence further improves image quality, reduces noise, and supports real-time parameter adjustments, thereby advancing the precision and effectiveness of thermophysical therapies. Currently, these techniques for treatment process monitoring are mainly based on offline data and are valuable for post-treatment evaluation, with limited studies integrating monitoring techniques into treatment systems. Further development of in vivo real-time temperature monitoring and ablation zone delineation technologies, and more importantly, the relationship between direct changes observed during the ablation and the follow-up damage and therapeutic outcome of biological system will enable more precise and accurate ablation procedures.
